# Vanishing Gastroschisis with a Favorable Outcome after a 3-Year Follow-Up: A Case Report and Literature Review

**DOI:** 10.1155/2020/8542087

**Published:** 2020-01-07

**Authors:** Elise Abi Rached, N. Sananes, I. Kauffmann-Chevalier, F. Becmeur

**Affiliations:** ^1^Department of Gynaecology & Obstetrics, Hautepierre, University Hospital, Strasbourg, France; ^2^Department of Pediatric Surgery, Hautepierre, University Hospital, Strasbourg, France

## Abstract

Vanishing gastroschisis (VG) is a severe complication of gastroschisis with a high mortality rate. We report here a case of VG with a favorable outcome after a 3-year follow-up. A 26-year-old primigravida woman was referred to Strasbourg University Hospital because her fetus was diagnosed with an isolated gastroschisis at 13-week gestation. The ultrasound evolution was marked by a progressive closure of the abdominal wall defect from 19-week gestation and the appearance of dilated intra-abdominal loops. The child was born with a closed abdominal wall except a small remnant at the level of the former gastroschisis orifice. Explorative laparotomy revealed extensive midgut atresia with only 50 cm of remaining midgut. A jejunocolic anastomosis was performed. The child is now 3 years old and has a favorable outcome with only 2 nights a week of parenteral nutrition. A total of 39 cases of VG type D from Perrone et al. classification are described in the literature from 1991 to 2019, among which 19 (48.7%) are alive at the time of publication but only 4 cases are described with a long-term follow-up of 3 years or more. This is the fifth case described with a favorable evolution after 3-year follow-up.

## 1. Introduction

Gastroschisis is an abdominal wall defect resulting in small intestine prolapse into the amniotic fluid without any protective covering membrane and variable degrees of malrotation. It is a rare congenital anomaly, but the incidence of gastroschisis has increased last few years [1, 2]. It is currently estimated at 5 per 10 000 births [3, 4]. Usually, gastroschisis is an isolated malformation, and affected neonates have a good outcome with an overall survival rate greater than 90% [5–7]. However, 17% of gastroschisis are complicated with intestinal atresia, perforation, necrotic segments, or volvulus and thus become complex gastroschisis [8, 9]. One of the most feared complications is the “vanishing gastroschisis” (VG). This happens when the abdominal defect is closing in utero in association with an extensive atresia of the small intestine and short-gut syndrome (SGS). The VG is thought to be the result of a vascular *in utero* accident. This could be explained by vascular injury to the developing intestine causing intestinal resorption; a strangulation and necrosis of the midgut by a narrow defect spontaneous closing or volvulus causing infarction, resorption, and closure of the defect [10]. Perrone et al. proposed in 2019 a new classification of closing gastroschisis [11]. Type D is defined as a completely closed defect with either a nubbin of exposed tissue or no external bowel. This is the category with the highest mortality rate around 70% [12] whereas it corresponds to our case. Only a few cases with a favorable issue are reported in the literature.

We report here a case of VG with a favorable outcome after a 3-year follow-up.

## 2. Case Report

**Figure 1:**
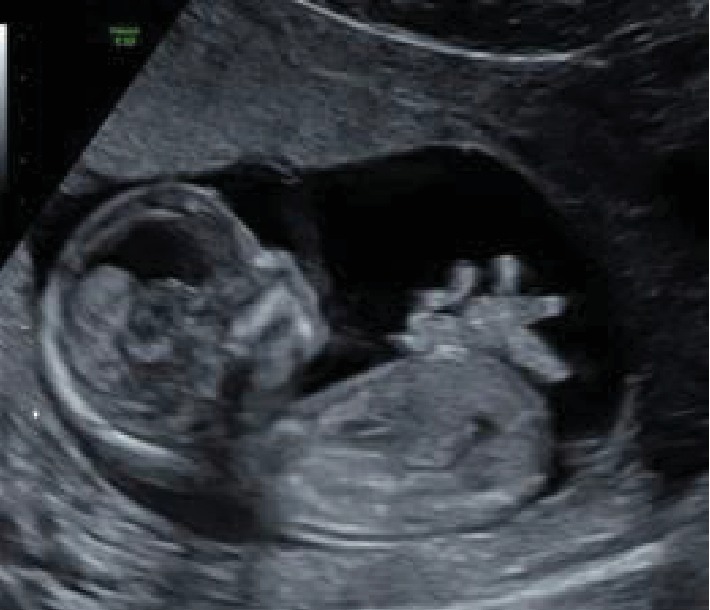
Gastroschisis at 13 weeks.

**Figure 2:**
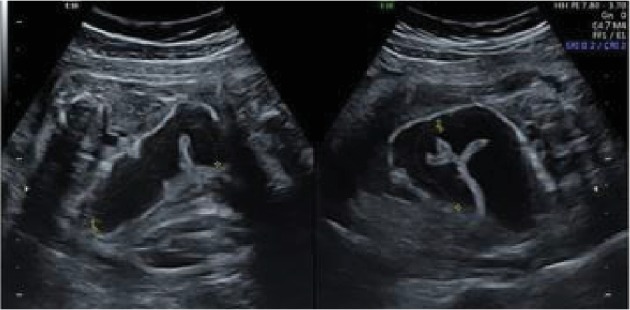
An important segmental intestinal dilatation at 32 weeks.

We report the case of a 26-year-old primigravida woman referred to Strasbourg University Hospital because her fetus was diagnosed with an isolated gastroschisis at 13-week gestation (Figure 1). At 19 weeks, the collar's size was narrow at 8 mm and there was a moderate dilatation of intestinal loops. At 24 weeks, the abdominal wall defect was not visible on the ultrasound and there was no intestine floating in the amniotic fluid. The small intestine inside the abdomen was very dilated suspecting intestinal atresia. A magnetic resonance imaging (MRI) is performed at 24 weeks and 30 weeks showing dilation of a small bowel loop on 8-10 cm, but it is impossible to measure the small bowel length remaining. At 34 weeks, ultrasound showed an important segmental intestinal dilatation (maximal length 32 mm of diameter) with conservative peristalsis (Figure 2). The amniotic fluid index was normal as the stomach size.

**Figure 3:**
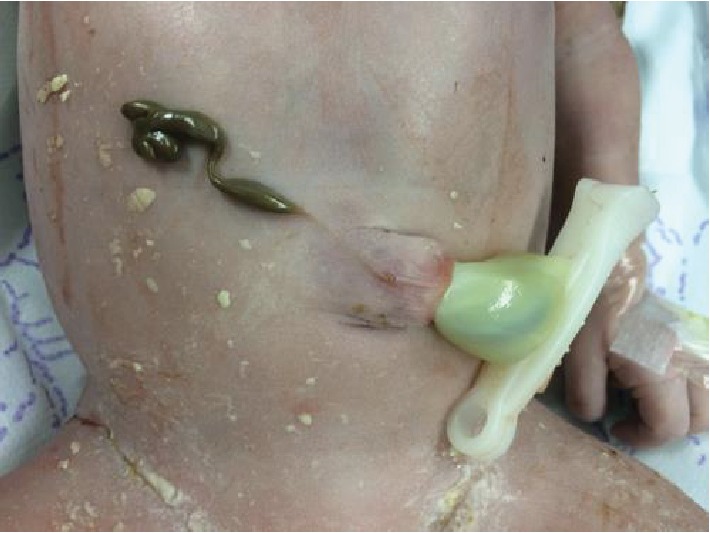
Paraumbilical remnant.

At 35 weeks of gestation, labor occurred spontaneously. A live male infant was delivered by normal vaginal delivery weighing 2560 grams with an APGAR score of 10 at 1 minute. There was no defect on the abdominal anterior wall except a small, grayish-brown paraumbilical remnant attached to a filiform axis crossing the abdominal wall (Figure 3).

**Figure 4:**
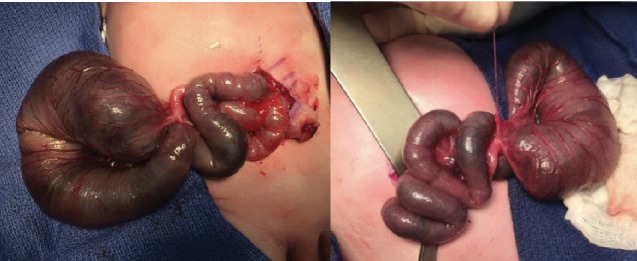
Explorative laparotomy, fibrous cord connected to paraumbilical remnant.

Abdominal X-ray with contrast product showed the presence of a voluminous blind intestinal loop of 3 cm in diameter and no passage in the colon. Surgical treatment by an explorative laparotomy was performed because of radiographic evidence of bowel obstruction. Exploration found 65 cm of a dilated small intestine downstream of the blind intestinal loop and atresia of the right colon. We found the same fibrous cord connected to the abdominal remnant and to the atresia zone (Figure 4). The remaining colon was filiform but permeable to the anus. Anastomosis ileocolic was performed after resection of a 15 cm necrotic small intestine. The total remaining small intestine length was 50 cm leading to SGS. The pathological examination of the abdominal remains confirmed the ileal origin. Parenteral nutrition was started with a central catheter, and oral feeding was started at 16 days postoperatively.

The evolution of the disease was marked by several sepsis starting points of the central catheter treated by antibiotherapy and catheter change. Oral feeding was progressively increased. At 2 years and 4 months, the parenteral nutrition was only 3 nights a week. Because of recurrent subocclusive episodes and dilation of distal bowel loops on imaging, a surgical treatment was decided. The small intestine was dilated up to 7 cm upstream of the permeable anastomosis: a new end-to-end anastomosis was performed. The small intestine length was 1.5 meters. At the age of 3 years, the boy was on parenteral nutrition only two nights a week. With growth, the child will probably be weaned from enteral nutrition in the months or years to come.

## 3. Discussion

VG is a rare complication of gastroschisis usually associated with a high rate of mortality closed to 70% [10, 13–16]. Even if they survive to SGS, the children with VG must face parenteral nutrition (PN) and its complications; some died from hepatic failure if they did not have the chance to receive a liver transplant [17, 18].

Perrone et al. proposed in 2019 a new classification from the analysis of 53 children with closing gastroschisis [11]. This classification reflects the expected long-term results. Type D represents only 8% of the patients.

A total of 39 cases of VG type D from Perrone et al. classification are described in the literature from 1991 to 2019 (Table 1), among which 19 (48.7%) are alive at the time of publication but only 4 cases are described with a long-term follow-up of 3 years or more. In 10 cases (25.6%), newborns had an explorative laparotomy and comfort cares only and died a few days after their birth. In 12 cases (30.8%), children had parenteral nutrition- (PN-) related complications from cholestasis to hepatic failure, and in 2 cases, children have benefited from hepatic transplant. The surgical management was dependent of the remaining length of small bowel, the presence of dilated bowel, or the presence of an ileocaecal valve [19, 20]. Some children have benefited a bowel lengthening procedure. This could be an autologous gastrointestinal reconstruction (AGIR), serial transverse enteroplasty (STEP), or longitudinal intestinal lengthening and tailoring (LILT) named Bianchi's procedure or an intestinal transplant.

In our report, antenatal ultrasound and fetal magnetic resonance imaging (MRI) failed to predict the remaining small intestine length. It seems difficult to get reliable prognostic factors to determine fetal outcome. Geslin et al. tried to evaluate prenatal ultrasound parameters as prognostic factors for complex and vanishing gastroschisis [21]. They report that the presence of intra-abdominal bowel dilation at the second or third trimester ultrasound was predictive for complex gastroschisis, with a cut-off value at the last examination of >19 mm. A small abdominal wall defect diameter was also predictive for complex gastroschisis, with cut-off values of <9.2 mm at T2 and <12.5 mm at T3. Robertson et al. analyzed 101 pregnancies complicated with gastroschisis. They demonstrated that the only statistically significant predictor of complex cases of gastroschisis was extra-abdominal bowel dilatation. Nevertheless, extra-abdominal dilatation was also present in antenatal ultrasounds of 44 neonates with simple gastroschisis. Other variables analyzed including intra-abdominal bowel dilatation, polyhydramnios, oligohydramnios, stomach dilatation, and stomach herniation were not statistically significant for predicting complex cases of gastroschisis [22]. In 2006, Garel et al. demonstrated in a few cases the interest of MRI to identify the level of the obstruction [23]. Matos et al. demonstrated that MRI had an interest in situations in which ultrasound has low sensitivity, such as maternal obesity, abdominal scarring, and oligohydramnios. Dilation larger than 17 mm and thickening of the loops of more than 3 mm can be related to high morbidity. To our knowledge, no study to date has evaluated the possibility of measuring the remaining small intestine length in case of VG which is a major prognostic factor [24]. The opportunity to have this information could help with prenatal counseling.

## 4. Conclusion

The VG is a rare and severe complication of gastroschisis with a high mortality rate due to SGS and to complications related to PN. Nevertheless, some children have a favorable outcome. Signs of closing gastroschisis in prenatal ultrasound should be carefully sought. Thereby, physicians can adapt prenatal counseling and prepare the parents for this complication and the need of multidisciplinary postnatal care [40].

## Figures and Tables

**Table 1:** 

First author	Case	Gestational age	Length of small bowel remaining	Type of surgery	PN	PN-related complications	Issue
Johnson [25]	1	38	0 cm (blind ending duodenum)	Explorative laparotomy only	NA	NA	Died at 4 days
Bromley [13]	2	36	0 cm	End duodenostomy	NA	NA	Died at 7 days
Bhatia [26]	3	34	25 cm jejunum	Jejunostomy and colonic mucous fistula and then closure of the stomas with anastomosis		Liver failure	Died at 18 months
Anveden-Hertzberg [18]	4		25 cm duodenum+jejunum	End jejunostomy		Liver failure at 8 months	Died at 10 months
Morris-Stiff [27]	5	36	10 cm jejunum	Explorative laparotomy only	NA	NA	Died a few days later
Kimble [28]	6	36	0 cm dilated (blind ending duodenum)	Explorative laparotomy only	NA	NA	Died at 7 days
Celayir [29]	7	36	25 cm jejunum	Jejunocolic anastomosis		Catheter-related sepsis	Died at 4 months
	8		10 cm jejunum	Explorative laparotomy only		NA	Died
	9		25 cm jejunum	Jejunocolic anastomosis. STEP at 6 weeks		Cholestasis	Alive at 4 months
Barsoom [10]	10	34	10 cm jejunum	LILT at 5 months		Liver failure at 8 months	Died
Ogunyemi [17]	11	32	15 cm jejunum	Jejunocolic anastomosis, intestinal transplantation at 53 months		Liver transplantation at 53 months	Alive at 4 years and a half
Davenport [30]	12	36	22 cm jejunum	Jejunostomy and mucous fistula, LILT at 5 and 12 weeks with jejunocolic anastomosis	Weekly parenteral infusion of electrolyte	Liver transplantation at 12 months	Alive at 2.5 years old
Basaran [31]	13	35	30 cm jejunum	Jejunocolostomy		Cholestasis	Died at 2 months
Winter [32]	14	35	17 cm jejunum	Jejunocolic anastomosis and LILT and then bowel transplantation			Alive at 32 months
Sandy [33]	15	35+ 5	30 cm small bowel	Jejunocolic anastomosis. STEP at 30 months	Daily	Cholestasis	Alive at 37 months
Vogler [12]	16-17		10 cm small bowel	Explorative laparotomy only	NA	NA	Died a few days later
	18		23.5 cm small bowel	Jejunocolic anastomosis, STEP at 6 weeks	Daily	Cholestasis	Alive at 4 months
Foucher [14]	19	31 + 5	0 cm (blind ending duodenum)	Explorative laparotomy only		NA	Died at 5 days
Houben [34]	20	32	15 cm jejunum	Jejunocolic anastomosis		Liver failure	Died at 9 months
Buluggiu [35]	21	38	45 cm jejunum	Jejunostomy, colostomy. Anastomosis one month later. Bianchi's procedure modified by Aigrain at 5 months	Stopped at 14 months		Alive at 25 months
Khalil BA 2010 [36]	22	36	30 cm jejunum	Bowel tube stomas, LILT at 6 months		NA	Alive at 2 years old
	23	33	20 cm jejunum	Jejuno-colic anastomosis		Liver failure	Died at 4 months
Lawther [37]	24	35	47 cm jejunum	Small bowel stoma and colonic mucous fistula. Closure of the stomas at 3 months. Revision of anastomosis at 5 months		Liver failure. Catheter-related sepsis	Died at 10 months
Dahl [38]	25	38 + 4	120 cm small bowel	End-to-end anastomosis		NA	Alive at 21 months
Kumar [39]	26	>37	0 cm (blind ending duodenum)	Explorative laparotomy only		NA	Died a few days later
	27	33 + 1	20-22 cm jejunum	STEP and jejunocolic anastomosis		NA	Transferred to transplant center
	28	33 + 1	13 cm jejunum	Explorative laparotomy only		NA	Died
	29	35 + 1	7-8 cm jejunum	Jejunocolic anastomosis		NA	Transferred to transplant center
Wood [40]	30	36	30 cm jejunum	Tube stomas. AGIR at 5 days	Stopped at 6 months		Alive at 3 years old
	31	35	20 cm jejunum	AGIR	4 days a week		Alive at time of publication
	32	33 + 5	20 cm jejunum	Currently undergoing active tissue expansion	NA	NA	Alive at time of publication
Dennison [15]	33	33	18 cm jejunum	Jejunostomy	NA	NA	Died at 28 days
Abdel-Latif [41]	34	>37	30 cm jejunum and 40 cm ilium	End-to-end anastomosis and double barrel colostomy	NA	NA	Alive at 30 days
Ponce [42]	35	32+ 5	27 cm jejunum	Jejunocolic anastomosis, LILT at 7 months	Daily		Alive at 7 years old
Perrone [11]	36-39	(33 + 5-35 + 6)	37 cm small intestine (mean)	3 (1-4) abdominal operations required (median with range)			One died. 3 alive at time of publication
Abi Rached	40	35	50 cm jejunum	Jejunocolic anastomosis, revision of anastomosis at 28 months	2 nights a week		Alive at 3 years old

AGIR: autologous gastrointestinal reconstruction; LILT: longitudinal intestinal lengthening and tailoring; NA: not applicable; PN: parenteral nutrition; STEP: serial transverse enteroplasty.
